# Conducting two evidence syntheses in six weeks – experiences with and evaluation of a pilot project

**DOI:** 10.1186/s12874-024-02334-y

**Published:** 2024-09-16

**Authors:** Heather Melanie R. Ames, Hege Kornør, Line Holtet Evensen, Ingeborg Beate Lidal, Elisabet Hafstad, Christine Hillestad Hestevik, Patricia Sofia Jacobsen Jardim, Gyri Hval

**Affiliations:** The Norwegian Institute of Public Health (NIPH), PO Box 222, Oslo, Skøyen 0213 Norway

**Keywords:** Evidence synthesis, Systematic review, Lean, Workflow

## Abstract

**Background:**

Evidence synthesis organisations are trying to meet commissioners’ needs for rapid responses to their evidence synthesis commissions. In this project we piloted an intensive process, working to complete evidence syntheses within six-weeks, rather than the standard lead time of 4–6 months. Our objective was to explore how researchers experience working intensively, identify barriers and facilitators, and determine how a more intensive approach to evidence synthesis could be more systematically introduced in the future.

**Methods:**

In a pre-planning phase, an intensive work group was established, and two commissions were selected for this pilot project. The evidence synthesis process was divided into two phases: planning and intensive. The planning phase, involved scheduling the intensive phase, exploring new digital tools, and identifying peer reviewers. The intensive phase encompassed the entire evidence synthesis process. Two review teams were formed, each with a team lead supported by a process lead and leadership contact point. Throughout the project, teams engaged in reflective meetings to evaluate and adjust processes as needed.

**Results:**

During the planning phase, teams identified significant uncertainties regarding scopes, research questions, and inclusion criteria. To address this, they engaged with commissioners earlier than originally planned, clarified these aspects, and prepared protocols. Despite some minor deviations from the original plan, both reviews were completed on schedule, with one team expanding their scope due to the absence of eligible studies. Teams operated flexibly, held regular meetings, and found the process seamless due to fewer interruptions. Machine learning tools facilitated rapid study selection. The process lead role, created to guide and evaluate the project, proved beneficial, providing structure and support, although clearer role delineation with the leadership contact point could have improved efficiency.

**Conclusions:**

Overall, the intensive process fostered focus and productivity, allowing teams to manage short-term deliverables effectively. The researchers preferred working intensively with one evidence synthesis over being involved with many projects at the same time. They felt that time use was more effective, and they were able to complete the tasks in a focused way. However, there are several implications that should be considered before implementing an intensive approach in future evidence syntheses.

**Supplementary Information:**

The online version contains supplementary material available at 10.1186/s12874-024-02334-y.

## Introduction

### Rationale for implementing the intensive team pilot

Evidence synthesis in general is under pressure to reduce lead time, i.e., the entire period between receiving the commission for an evidence synthesis and the moment a complete synthesis is delivered to the commissioner. One approach in response to the expectations of reduced lead time is to carry out a rapid review which can be done in a very compressed period, in some cases even within 24 h [1]. The disadvantage of the rapid review approach, however, is that it usually implies major methodological shortcuts to complete the project on time.

More recently, several providers of evidence syntheses, such as Bond University [2, 3] and the Cochrane Effective Practice of Care group [4, 5] have developed strategies to deliver full evidence syntheses in two weeks.

The Cluster for Reviews and Health Technology Assessments at the Norwegian Institute of Public Health (NIPH) is the largest scientific community in Norway specializing in evidence syntheses. The cluster delivers a range of evidence synthesis products commissioned by Norwegian health and welfare authorities, as well as the regional health trusts. We tailor the evidence syntheses to the commissioners’ needs, including the type of research question, level of detail, use of resources, and delivery time. For instance, our systematic reviews of effects of interventions are delivered in averagely six months from the date of commission, with 652 work hours spent. The cluster’s standard evidence synthesis team structure by 2022 was that each researcher was a member of at least two teams simultaneously, interchangeably with a team leader or team member role. Team leaders reported to a leadership contact point.

The last few years, a key focus of the cluster has been on how to reduce the lead time for our commissions without compromising methodological quality. To this end, the cluster has made some innovative efforts, such as a lean-inspired [6] project to improve workflows, and a machine learning team was established to evaluate tools to speed up the review process [7–10]. More recently, the cluster’s researchers have started to engage in sequential project processes where they work more intensely with one evidence synthesis at a time rather than carrying out two or more simultaneously [11–14]. Although not systematically evaluated, leadership has recognized that this more intensive approach could be successful and have other positive knock-on outcomes such as providing a more predictable planning horizon for project delivery dates and employee time use.

## Objective

Our objective was to explore how researchers experience working intensively, identify barriers and facilitators, and determine how a more intensive approach to evidence synthesis could be more systematically introduced in the future.

## Methods

One of the cluster’s directors (HK) took the initiative to design and pilot an intensive evidence synthesis process and established the “Intensive work group” for that purpose in January 2022. Eligibility criteria for participation in the work group were minimum two years’ experience with evidence syntheses, motivation to explore new work processes and some previous acquaintance with machine learning tools. The work group consisted of eight experienced researchers and information specialists who had responded to a call for interest across the cluster. They met periodically between January and August (the pre-planning phase) to discuss various approaches and confirm interest in participating in designing and piloting the intensive evidence synthesis process.

In the work group meetings during the pre-planning phase, we prepared an overall approach to the intensive evidence synthesis process by making some preliminary decisions regarding time frames, methodological standards, resources, commissions to be used for the pilot, roles and evaluation process.

## Time frame and methodological standards

### Time frame

We divided the intensive pilot project into a planning phase and an intensive phase.

#### Planning phase

The planning phase was the period between the pre-planning phase and the intensive phase, scheduled from 1 September to 23 October. During this phase the teams scheduled the intensive phase with milestones and deadlines. The planning phase also allowed the review teams to come to agreement on how they were going to work together. The teams planned to use the normal in-house procedures to develop a machine learning plan for study selection in EPPI-Reviewer (21), a systematic review software. In addition, they were tasked with exploring different new digital tools available to us. Finally, the planning phase gave the teams enough time to identify external peer reviewers, and any content expertise, contact them and have them agree to a shortened timeline. For insight into this process please see our meeting notes in supplementary file 1.

#### Intensive phase

The intensive phase was scheduled from 24 October to 13 December. To allow for events out of our control we set the deadline at almost seven weeks. During this period, the entire evidence synthesis process was to take place, including preliminary discussions with commissioners to define scope, research questions and inclusion criteria. The process should also include writing peer-reviewed protocols and full reports, based on NIPH’s standard methodological requirements, as defined by the Cochrane Handbook [15] and other international guidelines.

### Resources

As the size of the intensive work group became greater than expected, we decided to establish two review teams, one for each of the two evidence syntheses to be entered into the pilot project in parallel. Each team consisted of a team lead (LHE and CHH, respectively) and two team members; one researcher (IBL and PJJS, respectively) and one information specialist (EH and GH, respectively). To accommodate the demands of this intensive pilot project we also created a new role: the process lead (see Roles section below). The team leads, team members and process lead were allowed to use 1–2 days a week each to define the approach to their assigned evidence synthesis (the planning phase), and at least 50% of their work time during the six week-period to carry out and complete their respective syntheses (the intensive phase). Also, we agreed that they could block their time and not take on any new projects during the planning and intensive phases. The leadership contact point (HK) scheduled one hour per week, mainly to supervise the process lead.

### Commissions

Eligible commissions had to be narrow in scope with an ability to set clear and decisive inclusion and exclusion criteria. The commissioners had to be open to participating in an intensive pilot and available to answer questions quickly to meet project deadlines. Finally, the commissioners’ deadlines should be compatible with the project time frame.

Two eligible commissions, one systematic review and one systematic scoping review, were agreed upon in August 2022. The scope of the systematic review was to investigate the effect of national and regional incident reporting systems on serious events in patient care [16–18]. The scope of the scoping review was to map research on health and care services for older immigrants [19]. For more detail about the commissioned evidence syntheses see supplementary file 2.

### Roles

**Fig. 1:**
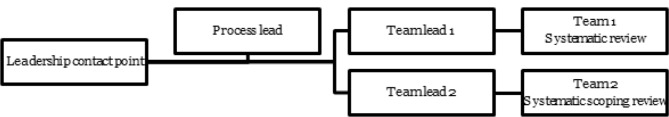
Intensive team structure

The project governance model consisted of a leadership contact point, a process lead, and two teams with one team leader and two team members, respectively (Fig. 1). The purpose of this model was to enable decisions to be made at the lowest possible level, thereby speeding up the synthesis process. It also allowed for a closer “lead” follow up of the intensive phase and more hands-on guidance around decision making and due dates.

Leadership contact point: initial contact with the commissioners to clarify their needs and to negotiate agreements between the parties. Throughout the intensive phase, the leadership contact point’s role was to support the process lead and the two teams with any matters concerning the projects’ frameworks.

Process lead: responsible for closely following up both review teams, having a good understanding of the content of both reviews, making sure deadlines were being kept and stepping in if a team needed additional resources. The process lead could also make some decisions regarding the implementation of the intensive phase process without conferring with the contact point.

Team leads: were responsible for one evidence synthesis each and coordinating their team members’ work. This included methodology but also communication with the commissioner.

Team assignment was done in a pragmatic way, as there was a natural split in interest and previous topic experience among the work group members.

### Evaluation process

At the end of each phase the intensive work group met for a reflective conversation meeting and discussed what had worked well, where the challenges had been and what we had learned/wanted to change in the process. Each work group member registered their time use throughout the project.

## Results

### Time frame and methodological issues

**Fig. 2:**
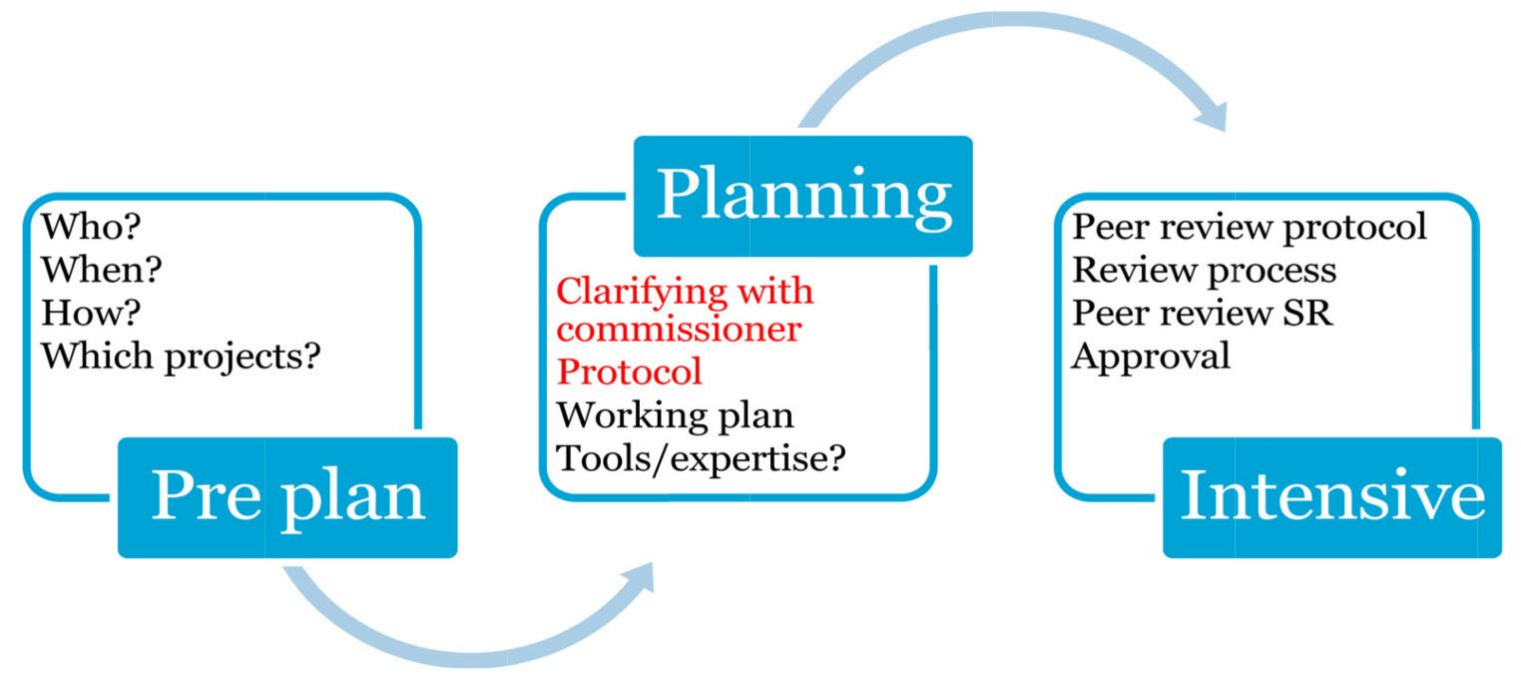
The three phases with tasks to carried out. Changes from original plan in red

Early in the planning phase, the teams realised that there were considerable unclarities regarding the scopes, research questions and inclusion criteria for both the reviews. They felt that it was essential to spend time with the commissioners discussing their needs and that these discussions could not wait until the intensive phase, as originally determined by the intensive work group. Therefore, the teams deviated from the original plan and clarified scope, research questions and inclusion criteria with commissioners and prepared the protocols for peer-review during the planning phase, as described in Fig. 2.

The time frames for the planning and intensive phases remained unchanged.

The timeline for the intensive phase entailed every step of the evidence synthesis process, from protocol submission to full reports ready for publication (Table 1).

**Table 1:** Timeline for the intensive phase

Date	Task
24.10	Intensive period starts: Protocol completed and sent to internal peer review with deadline 26.10. Not sent to external peer reviewers as subject specialists were involved in writing the project plan.
26.10	Project plan back from peer review
27.10	Search run
28.10	Project plan completed and sent for approval. Systematic review protocol registered in Prospero.
31.10	Screening and study selection start
04.11	Finalize including studies
07.11–09.11	Data extraction
10.11–18.11	Analysis (GRADE)
18.11	GRADE and Risk of Bias completed
21-30.11	Report writing
30.11	Send to internal and external peer review with deadline 05.12
05.12	Report back from internal and external peer review
07. 12	Report completed and sent for leader approval
13.12	Report approved and ready for publication

The two reviews were completed as scheduled by 13th December (please see Supplementary file 3). However, the systematic review concluded early (on 3rd November), as no eligible studies were identified. Thus, the systematic review team went on to expand the scope of the work and deliver a second report. Based on feedback from the commissioner and the topic expert, the team decided to include a description of some of the excluded studies that came close to the original inclusion criteria.

### Work process

All team members were flexible if there was a need to spend an extra day or swap days to work with the review. The teams received a weekly email on Mondays with the week’s goals from the process lead, had joint weekly status meetings with the process lead to inform each other and discuss status and challenges on Wednesdays and team leads submitted a weekly summary to the process lead on Fridays. Internal and external peer reviewers and commissioners were also given short deadlines as scheduled in the timeline.

Teams found that the planning and conducting of the reviews went seamlessly as there were fewer people involved. They were able to accomplish a lot in a day due to the limited number of interruptions. This intensive form of working gave more room to concentrate and focus on the tasks at hand. Daily update meetings helped keep the team on track and the weekly Monday emails from the process lead outlining the weeks tasks gave the team a concrete goal to achieve by Friday.

It was very beneficial to have two teams working through their commissions at the same time. They were able to discuss their challenges and solve problems together. The short timeline made teams make more pragmatic decisions around, for example, when to stop screening for grey literature or the length of certain sections of the report/how much we would offer to deliver.

### Tools

The machine learning functions in EPPI-Reviewer (21) are integrated as standard methods in the study selection process at NIPH, and three of the team members were experienced users of these functions. The teams used automatic text clustering, the Cochrane RCT classifier, Priority screening and OpenAlex in EPPI-Reviewer (21) in both reviews (Table 2).

**Table 2:** Machine learning functions that were used

Function	Description
The Cochrane RCT classifier	A tool to automatically identify and classify randomized controlled trials (RCTs) within large datasets of medical literature. It uses natural language processing (NLP) and machine learning algorithms to scan articles and determine whether they meet the criteria for RCTs, to speed up the study selection process
Priority screening	A tool that helps in the systematic review process by prioritizing which studies to screen first. Using machine learning algorithms, it assesses and ranks studies based on their relevance to the research question or topic of interest based on the researchers include/exclude decisions. This ensures that the most pertinent studies are reviewed early in the process, enhancing efficiency and effectiveness of the study selection process.
OpenAlex	A comprehensive, open-source dataset that provides structured information about scholarly publications, authors, institutions, and other related academic entities. It uses machine learning to organize and link various academic resources, making it easier to explore connections and trends in research. This tool is valuable for bibliometric analysis, research discovery, and enhancing the accessibility of scholarly information.

Both teams experienced that machine learning functions worked as expected and allowed them to complete their study selection in less than a week. We are aware that these tools may have limitations. For instance, priority screening relies on extensive training data from researchers to perform effectively and may exhibit selection bias if the training data is not comprehensive or representative. To mitigate this bias, the working group reviewed several references together at the beginning of the study selection process to ensure alignment and clarify the patterns of inclusion and exclusion for the algorithm. OpenAlex may be subject to data source bias, where certain journals, institutions, or regions are over-represented in the dataset. This was addressed by conducting systematic searches across several databases.

The teams were also tasked with exploring other machine learning or digital tools that could be useful in meeting our synthesis objectives, such as those within SR Accelerator [6] and a geoparser [19].

We also explored other digital tools that could be useful in meeting our synthesis objectives. During the planning phase, the whole intensive work group attended two workshops hosted by Bond University on functions designed to support the development of search strategies. These functions were the text frequency analyser (WordFreq), translation of search strategies between databases (Polyglot) (22), removing duplicate references (Deduplicator) and search refiner (SearchRefiner) (23), all which all of which are available at the Systematic Review Accelerator website [6].

When our librarians went on to explore WordFreq and SearchRefiner to develop the searches for our two reviews, some challenges arose. For the scoping review, we were unable to find seed articles that covered all aspects of the inclusion criteria (population, intervention and outcome) as the review question was so broad. This was despite the fact that we had a very large number (more than 60) of possible seed articles. For the systematic review, we had very few articles that were close to the inclusion criteria and none that explicitly met the inclusion criteria (the systematic review identified no relevant studies). After discussion, it was decided that the review questions we were working with were not ideal for testing these tools and that the searches would be developed as normal by the librarians. However, the librarians in the project used WordFreq and SearchRefiner on some chosen articles that met the inclusion criteria of specific PICO components that were important to include in the search strategy. They found that the SR Accelerator tools had a user-friendly interface, but it was still necessary to supplement with terms found through standard practice.

To identify which countries the studies originated from, the scoping review team originally planned to use a geo parsing tool [20] but decided not to follow through on this for two reasons. The first being that we could not find an easily accessible version to use that would not require a lot of learning time. The second being reflections and worries the team had about how geo parsing would work on the types of studies they were going to include. Since the research question was on immigration and migration the team was unsure if the studies would be correctly classified.

In the end, none of the new digital tools we explored ended up being fit for purpose for our review questions.

### Expertise and peer reviewers

The teams identified content experts within NIPH and involved them in writing the review protocols. Therefore, it was decided that the protocols only needed internal peer review.

One internal and two external peer reviewers were recruited during the planning phase. The internal peer reviewer was an expert in evidence synthesis methodology, while the two peer reviewers were content experts.

Peer reviewers are an important part of our evidence synthesis process. However, they can represent a risk when tight deadlines are involved, as in intensive evidence synthesis processes. We were very clear with the peer reviewers around our deadlines, however, in the scoping review there was a few days’ delay due to an external peer reviewer.

Peer reviewer feedback also influenced the timeline. In agreement with the commissioner, the teams were to keep the discussion sections brief. Peer reviewers had the most comments on the discussion sections of both manuscript drafts. They felt that we had not covered the relevant discussion areas and that the discussions themselves were too thin. Teams also felt that in the future we should be more specific about where peer reviewers’ comments could be taken on board and which parts of the report or inclusion criteria could not be changed. It was a challenge for commissioners, experts and peer reviewers to work with the higher tempo of these projects and they also had to change the way they were working with us. In most cases this was successful, but some delays were associated with this communication.

### Resources

The systematic review team had 600 h to answer their commissions. They spent 493,5 h to conduct and write up the systematic review with no included studies and the extra report describing some of the excluded studies that came close to the original inclusion criteria.

The scoping review team did not have a limited number of hours. They spent 491 h to complete the commission.

There were 191 h spent on the intensive work group across all members involved. These hours covered the planning process, exploration of new tools to complete the commissions, reflection meetings and the process lead’s time to follow up the teams and write a process evaluation report.

The teams spent approximately one day a week in the period scheduled to plan for the intensive phase (1st September to 23rd October 2022). The teams met frequently, in person or digitally. The teams felt that the planning phase could have been completed faster if it had been more intense, that is, they could have spent more working days per week over fewer weeks. However, this would have required the commissioner to set aside larger chunks of time to sit down and work through the inclusion criteria and research question with the teams.

During the planning phase, the teams agreed to reserve three full workdays in their calendars for every week of the intensive phase. During the intensive phase, it became clear that blocking whole working days in the calendar was not feasible. Team members had to accommodate other projects and meetings as well as be available for each other. The teams also felt that they could not let the project sit for several days and ended up working on the projects almost every day rather than just the allocated days. However, blocking of whole project days did protect team members’ time and discouraged others from calling them into meetings. Some team members indicated that it was hard not to work overtime as this type of intensive work blended with other projects and did not fit well with the current system of working on multiple projects at once.

A few team members were balancing multiple projects during the intensive phase. These other projects had to be flexible and adjust. If they were not, then the team members felt it was difficult to follow up both projects as they were so focused on the intensive pilot project that it took some time to reengage. It was also stressful for these team members to have multiple projects that had to be delivered at the same time.

They focused on the tasks at hand and breaking the review process down to manageable and short-term deliverables. Members felt that they used about 70% of their time on the intensive pilot project and this was necessary to be focused and meet the project deadlines.

### Roles

The process lead role is something we developed for this project. It was intended to track the implementation of the pilot and evaluate the process. It was also intended as a role that would relieve the leadership contact point from the task of following up the two teams very closely to provide process guidance, help solve simpler methodological challenges and help the teams achieve the rapid pace of conducting these reviews. The team leaders in both intensive projects maintained a normal team leader role with full control over what was happening in their team.

The intensive work group discussed what knowledge, background and skills a process lead should have. We felt that the person in this role should be very organized and effective at managing time. They should understand the systematic review process and be able to help solve methodological challenges. They need to provide a sense of safety to team members and be supportive in caring for those involved.

Both team members and the process lead felt that this new role was beneficial for this pilot project. It provided structure and guidance to a fast-moving process, keeping goals and deadlines clear and maintaining focus on the task at hand. The main role of the process lead in this intensive pilot project was also to conduct the evaluation and have the teams reflect on the process as well as keep them on track for delivery. Alternatively, this role could be built into team lead positions with clear guidance on process goals, planning and implementation if an evaluation of the process is not needed.

We would have benefited from an even clearer role division between the process lead and the leadership contact point with scheduled meetings and check in questions. The process lead and leadership contact point had agreed to have regular meetings throughout the project, however, these did not happen as planned. Reflecting on the pilot we also realized that we should have discussed and parsed the process lead’s and leadership contact point’s respective tasks and responsibilities to avoid confusion amongst the review teams. These were quickly clarified during the project but led to some momentary confusion concerning, for example peer reviewers and draft report approval.

Finally, the two teams engaged with and used the process lead and leadership contact point differently. The systematic scoping review team had a simpler research question that followed a straightforward process. This team leaned on the process lead most near the end of the project for report finalization but were independent for most of the intensive phase. The systematic review team had a more complex research question and ended up with no studies meeting their inclusion criteria. Because of the need to renegotiate with the commissioner and develop a new report to deliver, the process lead and leadership contact point worked much more closely with this team as the project became more tailored to the commissioner’s needs. This team equally drew on the process lead for help in finalizing their report.

## Discussion

The intensive project team enjoyed the pilot process and believed that working this way in the future could be a good solution for some of our projects. They found that this approach to conducting evidence synthesis allowed for incremental progress that was more structured, adaptive, and efficient than the traditional linear method of conducting systematic reviews. However, the intensive work group came to agreement on several reflections around implementation that we feel are important to consider if implementing an intensive approach to conduct an evidence synthesis.

In future similar initiatives, the pre-planning phase does not need to be implemented. This phase can be covered by leadership as they decide on commissions and put together review teams. The process lead position may not need to be implemented in future projects with experienced team leads without a process evaluation component.

This intensive way of conducting evidence synthesis allows for more predictability of when projects will start and finish and employee time use (who will be available when). Team members believed that it would be feasible to plan the next project while working in the intensive phase of another.

It is important that all team members are involved in the planning phase to facilitate an in-depth understanding of the project and research question. During this phase, team members need to focus on the project plan and on how they are going to work together. Researchers should only work on one systematic review at a time and have limited involvement with other commissions during the intensive phase. In general, team members felt that having three team members made them vulnerable to delay due to potential illness or other projects.

If protocols are going to be sent to external peer review, then this needs to be considered in the timeline. One option would be to extend the intensive phase to 8–9 weeks if both project plans and final systematic reviews are being sent to peer review to accommodate potential delays. The review team and the commissioners should come to an agreement beforehand on how to proceed if one of the peer reviewers is delayed (proceed with only 1 external review or wait for the second). Finally, both the commissioners and the peer reviewers need to be aware of and agree to the more intensive timeline and the deadlines that this will entail. A longer period of time may also be needed for projects that have a large number of topic experts involved and/or require more reflection around difficult questions or topics.

**Fig. 3:**
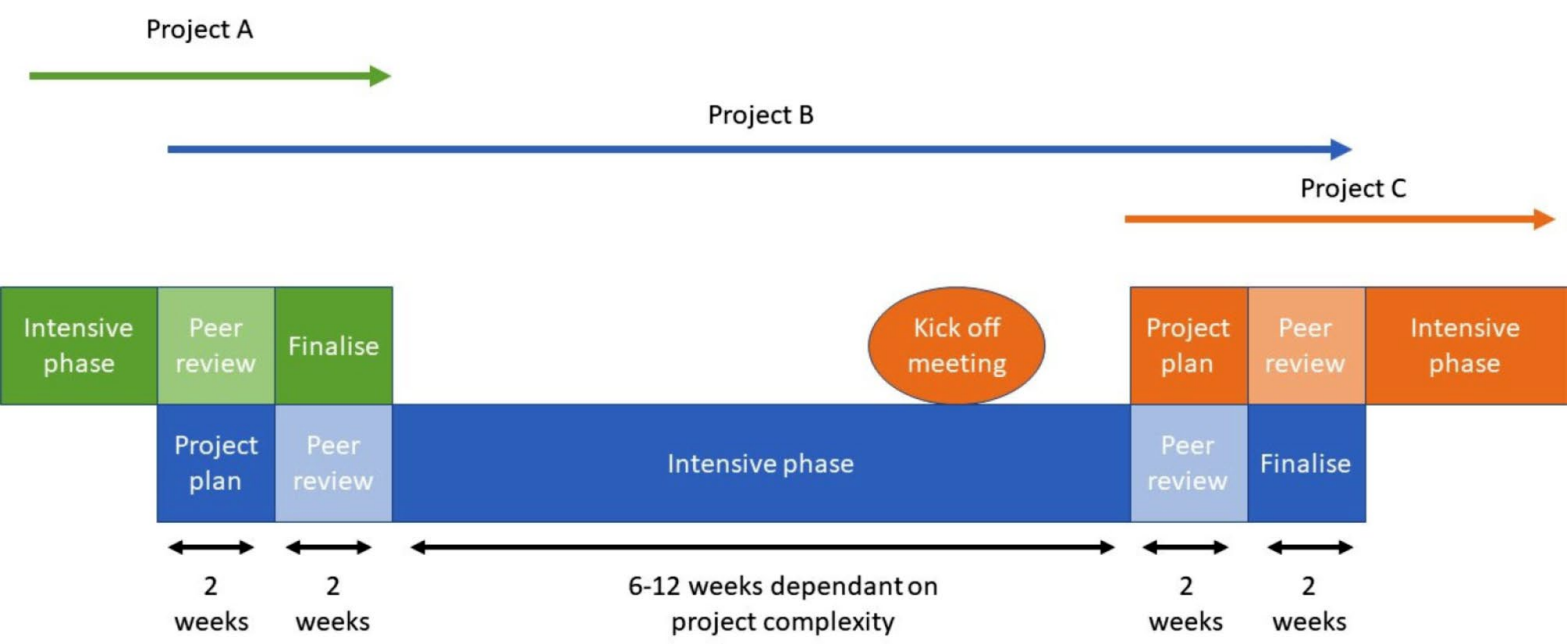
Conceptual option 2 for future implementation

The intensive pilot project team discussed to conceptual options for future implementation. The first option was a parallel option where one systematic review would be developed while the other was carried out. The second option would see giving external peer reviewers the normal 2 weeks to review a project plan or final report with development of the next project being done mostly during this time. The kick-off meeting for the upcoming project would happen during the intensive phase of the ongoing project. However, detailed question development, inclusion criteria, finalizing the search and writing the project plan would happen mostly in the two weeks while the previous end report is out to peer review. The first kick off meeting could be short and digital. This meeting would be used to discuss the background behind the commission, what the report will be used for and clarify any early uncertainty around the research question. The second meeting would be a longer face to face meeting (up to 1 day) during the early stage of the planning phase, where the research team would talk about the working process and deadlines, set expectations around communication, discuss the various types of evidence synthesis that could meet the commissioners needs and work on defining the question and the inclusion criteria. See Fig. 3.

No matter which approach is taken, we feel that the final delivery date should not be confirmed until the team knows the number of studies that meet their inclusion criteria. At this point the team timeline and resources can be re-evaluated dependent on the number of included studies. If no studies are identified a revised plan can be agreed upon with the commissioner.

Working this way is very intensive. Leadership will need to ensure that a team member is not involved in more than one intensive evidence synthesis at a time. Team and personal chemistry, systematic review experience and working styles need to be taken into consideration when putting an intensive team together. Co-workers need to trust each other and work well together to complete the project. Leadership needs to be aware of the weaknesses of the intensive phase such as the lack of time for reflection during the project and the short timeline for extra support if needed (i.e., for analysis).

In future projects, it will be important to keep exploring other digital tools and solutions that can be helpful in speeding up the review process without compromising quality. This intensive form of conducting systematic reviews depends on team knowledge of digital tools and machine learning to support the process. This project should also be piloted on a full systematic review of effect with a meta-analysis and/or other more complicated knowledge synthesis products.

## Conclusions

Our pilot project successfully demonstrated that systematic reviews can be completed intensively within six weeks. Overall, the intensive process fostered focus and productivity, allowing teams to manage short-term deliverables effectively. The researchers preferred working intensively with one evidence synthesis over being involved with many projects at the same time. They felt that time use was more effective, and they were able to complete the tasks in a focused way. However, there are several implications that should be considered before implementing an intensive approach in future evidence syntheses.

## Electronic supplementary material

Below is the link to the electronic supplementary material.

Supplementary Material 1

Supplementary Material 2

Supplementary Material 3

## Data Availability

All data generated or analysed during this study are included in this published article and its supplementary information files.

## Abbreviations

HTA: Health technology assessment
NIPH: Norwegian Institute of Public Health
