# Supplementary material for: Conducting two evidence syntheses in six weeks – experiences with and evaluation of a pilot project
Source: BMC Med Res Methodol. 2024 Sep 16;24:208. doi: 10.1186/s12874-024-02334-y (PMC11403876; doi:10.1186/s12874-024-02334-y)
Supplement: Supplementary file 1 — Supplementary Material 1 [file 12874_2024_2334_MOESM1_ESM.docx]

# Supplementary file 2_ Description of the systematic review and scoping review projects

#### The systematic review

The Norwegian Ministry of Health and Care Services commissioned NIPH to conduct a systematic review to investigate the effect of national and regional incident reporting systems on serious events in patient care.

The objective of our review was to inform an expert group (Expert group on reporting system for severe adverse events in the health and care services) in their evaluation of the incident reporting systems of Norwegian Board of Health Supervision and The Norwegian Healthcare Investigation Board.

All providers of health and social services must work systematically to reduce the risk of adverse events resulting from the provision of services or a lack of provision. Nevertheless, unwanted events of varying severity do occur. It is estimated that an adverse event affects one in ten patients in Norwegian hospitals. Norwegian health service providers are obliged to report serious incidents to the Norwegian Board of Health Supervision (the Board of Health) and The Norwegian Healthcare Investigation Board (NHIB). Research on the effect of general national and/or regional incident report systems on patient safety outcomes would be highly relevant to support the work of the expert group evaluating the Norwegian system.

The team produced, a systematic review and rapid mapping review (13-15).

#### The systematic scoping review

The Norwegian Ministry of Health and Care Services commissioned NIPH to conduct a scoping review on research on health- and care services to older immigrants. This is because the Norwegian government is working on a reform to ensure older people the possibility to live in their own home for as long as possible. The main aim of the reform is to make it safe for the elderly to stay at home for longer and ensure sustainability through better planning, strengthened prevention and more targeted services that promote coping. However, seniors in Norway are a heterogenous group. Seven per cent of all seniors in the country are immigrants originating from 197 different countries. We lack knowledge about many groups of older immigrants in Norway, but what we do know from Statistics Norway's survey of living conditions among immigrants is that they generally report poorer self-reported physical and mental health than the general population. Reviews of immigrants' use of municipal health and care services also show that older immigrants use the services to a lesser extent than the rest of the population. Older immigrants do not necessarily have the same needs and preferences for health and care services as the majority population. Cultural and social factors influence health behavior and patients' opportunity, desire, and ability to access and use health and care services.

There is a great need to adapt the services to a complex and heterogeneous population to ensure an equitable service for all. However, we lack knowledge about the existing research in the field and in what areas research is lacking. Thus, the review’s objective was to map available research on older immigrants, family caregivers and healthcare personnel’s experiences with and perspectives of health and care services for older immigrants, as well as interventions to adapt health and care services for older immigrants.

The team produced a systematic mapping review (16).
