# Supplementary material for: Conducting two evidence syntheses in six weeks – experiences with and evaluation of a pilot project
Source: BMC Med Res Methodol. 2024 Sep 16;24:208. doi: 10.1186/s12874-024-02334-y (PMC11403876; doi:10.1186/s12874-024-02334-y)
Supplement: Supplementary file 2 — Supplementary Material 2 [file 12874_2024_2334_MOESM2_ESM.docx]

# Supplementary file 1_ The content of the planning meetings

#### Planning meeting 1 (31.08.22)

We decided which researchers were going to work on which projects. The team discussed team roles and expectations of the process. A focus of this discussion was being open to trying new things and new approaches. We also discussed what would be encompassed in the planning phase and which tasks would be completed as part of the intensive phase. Furthermore, we discussed what types of support or expertise we would need as part of the planning process and how often and for how long we would meet to work.

At this first meeting it was decided to meet as a group on Mondays to discuss process related challenges and planning. Teams would then meet separately to work on project specific planning at a time that was convenient to them.

#### Planning meeting 2 (07.09.22)

The goal of this meeting was to make a plan for the interim deadlines in the planning and intensive periods. It was decided that the methods section of the protocol would be completed as part of the planning phase. The focus here was on confirming the inclusion and exclusion criteria with the commissioner and deciding on the methods to be used in each project. We also decided to have a discussion with the commissioners about protocol and report format: what was necessary to include and what could be shortened or removed? During this week teams came to agreement about their internal structure and how they would delegate tasks. A team leader was named for each of the systematic reviews. We agreed on a loose outline for the intensive phase.

#### Planning meeting 3 (12.09.22)

During this meeting teams presented on their agreed upon team structure and how they had decided to block their time and work together during the intensive phase. Teams agreed to block at least two days a week in their calendars during the intensive phase. They also agreed to meet at the beginning of each working day to set daily goals and to meet at the end of the work period to summarize what goals had been achieved, further work needed and the next steps for the following week. It was also agreed that process lead (HA) would send out an email at the beginning of each week with the tasks for the week and what had been accomplished the week before. The whole group would meet on Wednesdays for a quick check in meeting to discuss barriers and any questions about process or their specific review. Team leaders would send a weekly summary to process lead (HA) on Fridays presenting the weeks accomplishments and what they would focus on in the upcoming week.

Alternative research plans were also discussed for example, what if no studies are found? Too many studies are found? Teams agreed to start identifying peer reviewers and other tools they would need. Finally, we decided on the internal delivery deadlines within the project.

#### Planning meeting 4 (19.09.22)

We continued to discuss the details around timelines and how we would work together during the intensive process. Each team presented an update on their review objectives and how the protocol was progressing.

#### Planning meeting 5 (10.10.22)

Each review team presented an update about their review objectives and protocols. Any challenges were discussed. Teams also discussed how they were going to involve the commissioner in the intensive process, for example, arranging a meeting already to discuss the studies that meet the review inclusion criteria. We discussed commissioner responsiveness and identification of peer reviewers. Finally, we discussed how we would adapt if a team member became ill or had to step out during the intensive phase.

#### Planning meeting 6 (17.10.22)

We started the meeting with each review team presenting an update of their review objectives and protocol and discussing any challenges. We then finalized how the teams and the group were going to work together during the intensive process. Finally, we had a conversation reflecting on the planning period and learning points so far.
