# Supplementary material for: Conducting two evidence syntheses in six weeks – experiences with and evaluation of a pilot project
Source: BMC Med Res Methodol. 2024 Sep 16;24:208. doi: 10.1186/s12874-024-02334-y (PMC11403876; doi:10.1186/s12874-024-02334-y)
Supplement: Supplementary file 3 — Supplementary Material 3 [file 12874_2024_2334_MOESM3_ESM.docx]

# Supplementary file 3_ Timeline for actual work outputs

*Project timeline and deadlines compared to actual work outputs for the systematic scoping review*

| Task plan | | Scoping review actual work outputs | |
| --- | --- | --- | --- |
| **24.10** | Protocol completed and sent to internal peer review | **24.10**  **27.10*** | Protocol completed and sent to internal peer review  Protocol sent to external review with a two-day deadline |
| **26.10** | Project plan back from peer review | **26.10**  **29.10** | Project plan back from internal peer review  Project plan back from external peer reveiw |
| **27.10** | Search run | **25.10**** | Database searches run |
| **28.10** | Project plan completed and sent for approval. | **31.10** | Project plan completed and sent for approval. |
| **31.10** | **Screening and study selection start** | | |
| **04.11** | Finalize included studies | **10.11** | We searched for relevant studies in grey literature and in OpenAlex parallell to data extraction from the included studies from the database searches |
| **07- 09.11** | Data extraction | **7- 18.11** | Data extraction and mapping of evidence base |
| **21-30.11** | **Report writing** | | |
| **30.11** | **Send to internal and external peer review** | | |
| **05.12** | **Report back from internal and external peer review** | | |
| **07. 12** | **Report completed and sent for leader approval** | | |
| **13.12** | Report approved | **14.12** | Report approved |

*As we collaborated with a topic expert on the project plan we did not plan to send it to external review. The internal reviewer did however mean that this was necessary and we decided to send it to an external reviewer according her feedback. ** The search was run before feedback from reviewers as we needed an overview of the approximate number of search hits to plan our work. We planned to adapt the search strategy according to feedback from reviewers if necessary.

***Supplementary table 3:*** *Project timeline and deadlines compared to actual work outputs for the systematic review*

| Task plan | | Systematic Review actual work outputs | |  |
| --- | --- | --- | --- | --- |
| **24.10 Protocol completed and sent to internal peer review *** | | | | |
| **26.10** | Project plan back from peer review | **25.10** | Protocol back from peer review | |
| **27.10** | **Search run** | | | |
| **28.10** | Project plan completed and sent for approval.  Systematic review protocol registered in Prospero. | **27.10** | Project plan sent to approval (approved on 31.10).  Registration in PROSPERO completed | |
| **31.10** | Screening and study selection start | **28.10** | Screening and study selection start | |
| **04.11** | Finalize included studies | **03.11** | Screening completed. No included studies | |
| **07- 09.11** | Data extraction | **04.11** | Meeting with commissioner to discuss further solutions (agreed on: a) finish deliverable 1 – empty systematic review, b) deliverable 2 – mapping include risk of bias-assessment of selected research excluded from systematic review***) | |
| **10-18.11** | Analysis (GRADE) | **04.11-09.12** | Report writing (deliverables 1 and 2) | |
| **18.11** | GRADE and Risk of Bias completed | **14.11** | Report (deliverable 1) sent to internal and external peer review | |
| **21-30.11** | Report writing | **28.11** | Report (deliverable 1) back from last peer reviewer | |
| **30.11** | Send to internal and external peer review | **06.12** | Report (deliverable 1) complete and sent for leader approval | |
| **05.12** | Back from peer review | **08.12**  **09.12** | Report (deliverable 1) approved and ready for publication  Report (deliverable 2) complete and sent to for leader approval | |
| **07. 12** | Report completed and sent for leader approval | **12.12** | Report (deliverable 2) approved and ready for publication | |
| **13.12** | Report approved and ready for publication |  |  | |

* Because a topic expert was involved on the commissioners’ side, no peer reviewers were involved in deliverable 2 (as per NIPHs quality assurance procedure for this product type).
